# Supplementary material for: Prevalence and predictors of suboptimal glycemic control among patients with type 2 diabetes mellitus in northern Thailand: A hospital-based cross-sectional control study
Source: PLoS One. 2022 Jan 18;17(1):e0262714. doi: 10.1371/journal.pone.0262714 (PMC8765659; doi:10.1371/journal.pone.0262714)
Supplement: S1 Questionnaire — (DOCX) [file pone.0262714.s001.docx]

**Questionnaire**

**Blood glucose □ Controlled □ Uncontrolled**

**Part 1 P**hysical examination and biomarkers

Weight ........................................... kg Height......................................... cm

Blood pressure.................................. mmHg

LDL-Cholesterol…………………………….…. mg/dL

HDL-Cholesterol………………………………. mg/dL

Triglyceride………………………………………. mg/dL HbA1C…………………………………………………mg%

**Part 2** General information

1. Sex □ Male □ Female

2. Age…………....……. years

3. Religion □ Buddhism □ Christian □ Islam

4. Ethnics □ Hill tribe □ Indigenous

5. Thai ID card □ No □ Yes

6. Education □ Non-education □ Primary school □ High school □ University degree

7. Occupation □ Unemployed □ Agriculturist □ Unemployed

8. Income (per years) ..............................................Baht

9. Debt □ Yes □ No

10. Marital status □ Single □ Married □ Ever married

11. Living with □ Aloneอยู่คนเดียว □ Husband/Wife □ Daughter

12. Kidney disease□ Yes □ No □ Not sure

13. Hypertension □ Yes □ No □ Not sure

14. How long have you been diagnosed as diabetes____________ years

15. Have you forget taking diabetes medication last week? □ Yes □ No

16. Have you forget taking diabetes medication last month? □ Yes □ No

17. Do you have any experiences or side effects from diabetes medication? □ Yes □ No

18. How do you handle your medical expenses? □ Universal Health Coverage □ Pay yourself

19. Have you had any foot ulcers? □ Yes □ No

20. Family history of hypertension and diabetes

| **History** | **Diabetes** | | | **Hypertension** | | |
| --- | --- | --- | --- | --- | --- | --- |
|  | **Yes** | **No** | **Not sure** | **Yes** | **No** | **Not sure** |
| Father |  |  |  |  |  |  |
| Mother |  |  |  |  |  |  |
| Grandfather |  |  |  |  |  |  |
| Grandmother |  |  |  |  |  |  |

**Part 3** Health behaviors

1. Smoking □ No □ Ever □ Yes ________ years

2. Alcohol use □ No □ Ever □ Yes ________ years

3. Exercise □ No □ Sometime □ Everyday

5. How did you get daily food? □ Self cooking □ Buying

6. What kind of rice do you eat? □ Non-sticky rice □ Sticky rice

7. Having tea □ No □ Sometime □ Regularly

8. Having coffee □ No □ Sometime □ Regularly

**Part 4** Stress assessment (ST-5) Please indicate your experience within two weeks

| **Feeling** | **No** | **Sometime** | **Often** | **Regularly** |
| --- | --- | --- | --- | --- |
| 1. Insomnia |  |  |  |  |
| 2. Lack of concentration |  |  |  |  |
| 3. Irritability |  |  |  |  |
| 4. Boring |  |  |  |  |
| 5. I do not feel like going out and meet people |  |  |  |  |

**Part 5** Assessment of knowledge about diabetes prevention and control

| **Items** | **True** | **False** | **Not sure** |
| --- | --- | --- | --- |
| 1. If a parent has diabetes, then will their child have diabetes in the future? |  |  |  |
| 2. Frequently urinating, weight loss, fatigue could be an early sign of diabetes |  |  |  |
| 3. People with diabetes are more likely to develop cataracts |  |  |  |
| 4. Eating too much sugar is the cause of the diabetes |  |  |  |
| 5. Being overweight raises risk for diabetes |  |  |  |
| 6. High blood pressure as a risk factor for diabetes |  |  |  |
| 7. Being more than 30 years of age is a risk factor for diabetes |  |  |  |
| 8. Diet control can reduce the risk of developing diabetes |  |  |  |
| 9. Regular physical activity can reduce the risk of developing diabetes |  |  |  |
| 10. Can diabetes be cured |  |  |  |

**Part 6** Assessment of attitudes towards diabetes prevention and control

| **Items** | **Agree** | **Neutral** | **Disagree** |
| --- | --- | --- | --- |
| 1. Diabetes is not a scary disease because everybody can be |  |  |  |
| 2. If you have diabetes, it will make fatigue and weakness unable to work. |  |  |  |
| 3. Diabetes mellitus is affecting only older |  |  |  |
| 4. Eating a lot of white rice on a regular no risk for diabetes |  |  |  |
| 5. If you are not diabetic, it is not necessary to control weight |  |  |  |
| 6. Natural sugars like those found in fruits and vegetables are not linked to diabetes risk |  |  |  |
| 7. If you do not take your diabetes medication as prescribed has no effect on treatment |  |  |  |
| 8. Obesity is the leading risk factor for diabetes |  |  |  |
| 9. If a parent no has diabetes, then will their child no have diabetes too. |  |  |  |
| 10. If diabetics can control their food intake. It is not necessary to go to the hospital to pick up medicines. |  |  |  |
